# Commitment to integrity and transparency in research

**DOI:** 10.1590/1518-8345.0000.3403

**Published:** 2020-10-05

**Authors:** Regina Aparecida Garcia de Lima, Evelin Capellari Cárnio

**Affiliations:** 1 Universidade de São Paulo, Escola de Enfermagem de Ribeirão Preto, PAHO/WHO Collaborating Centre for Nursing Research Development, Ribeirão Preto, SP, Brazil.

**Figure:**
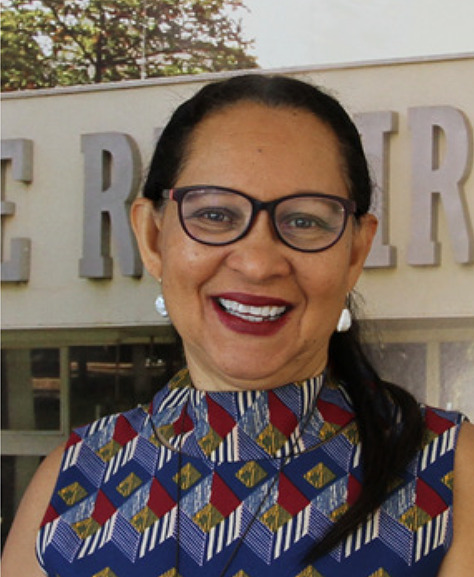

**Figure:**
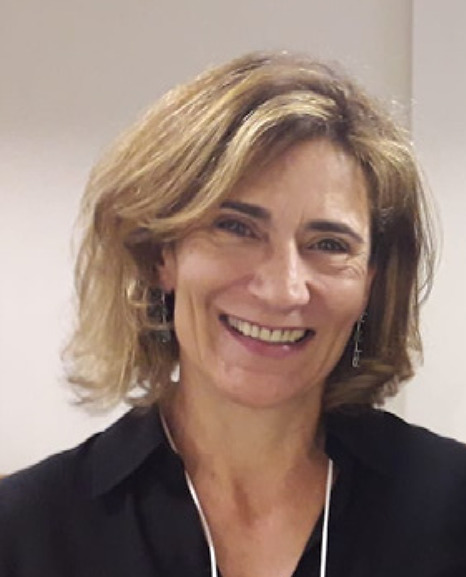

The Latin American Journal of Nursing (*Revista Latino-Americana de Enfermagem*, RLAE) aims to contribute to the advancement of scientific knowledge and professional practice in Nursing and other health areas through the publication of articles of high scientific and social merit that have followed the research integrity parameters and ethical codes of conduct recommended by the International Committee of Medical Journal Editors (ICMJE)^(1)^, the Committee on Publication Ethics (COPE)^(2)^, and the Code of Conduct and Best Practice Guidelines for Journal Editors^(3)^.

In accordance with good scientific dissemination practices and with the demands of Open Science, RLAE has revised some recommendations of its editorial policy, which have been incorporated into the instructions to authors and are highlighted below:

- The Journal now accepts for evaluation scientific manuscripts published on servers or national and international Preprints repositories recognized by the academic community. It also receives manuscripts in which the contents (data, program codes, and other materials) are available in data repositories recognized by the academic community, strongly encouraging this deposit;
- It adds the nominal identification of the Associate Editor who conducted the evaluation process at the time of publication of the article. This is the first step in opening the peer review process;
- It clearly describes that the criterion of scientific merit is a priority in the acceptance of manuscripts for publication, also considering the potential for advancing scientific knowledge in the theme resulting from the study; their contributions to the advancement of clinical practice and/or teaching and/or development of public health policies and/or future research; the scientific quality identified by the method and analysis employed; rigor and originality in the presentation of results; global relevance and interest.

The EQUATOR Network guides^(4)^ are adopted by RLAE in order to improve the quality and transparency of health research and help the author to present all the relevant and necessary aspects in the writing of scientific articles. It is noteworthy that the partial description of the results (omission of some outcomes), the omission of essential information about the method, the results and adverse effects of the therapy, incomplete description of the interventions, and poorly prepared abstracts are aspects that prevent the acceptance of manuscripts for publication. The guides must be used by the authors in the elaboration of their manuscripts and by the consultants and associated editors in their evaluation.

Among the various guides provided by the Network is CONSORT (Consolidated Standards of Reporting Trials), created in 1996 to guide authors to report the methods and results of randomized clinical trials. As it is considered the gold standard for clinical practice in health, randomized clinical trials were highlighted in the recommendations of the ICMJE^(1)^ for best practices and ethical standards in conducting and reports of research published in medical journals, in item III.L, whose specifications are required by RLAE.

The first topic dealt with in this item concerns the registration of clinical trials in public registries in order to prevent the selective publication of research results; avoid unnecessary duplication of research efforts; inform the community about planned or ongoing trials, and contribute to ethics committees by providing input for the approval of new studies under evaluation. The retrospective registration of a research protocol does not serve any of these purposes. The information on the number of the public registry of clinical trial studies at the end of the summary is a requirement of RLAE, as well as the presentation of the checklist and flowchart of the CONSORT guide.

CONSORT endorsement by medical journals has been one of the most widespread actions aimed at improving the integrity levels of randomized trial reports. The evidence, however, shows that despite modest improvements, when CONSORT is supported by journals, the quality of clinical trial reports remains insufficient^(5)^.

The sending of the CONSORT checklist by the authors is not always a guarantee that the CONSORT items were effectively satisfied. Among the reasons for the presence of inconsistency between the informed and the defendant, two deserve to be highlighted: it is possible that the authors are not aware of the CONSORT requirements or that, despite their efforts to comply with the requirements, the way the items are presented does not allow for interpretation at the level of detail required. Misinterpretation of CONSORT is a major concern, as it means that essential information about the conduct of the study is poorly communicated. Second, difficulties can lie with consultants and editors. It is possible that they feel confident about the quality of the reports only because of the presence of a complete checklist. Among the possible solutions and in an effort to make the most of requiring checklist submission, journals should consider clarifying their position on whether the complete checklists or, at least the main items on the checklists verification, should be examined by editors or reviewers or even by trained editorial assistants^(6)^, a position assumed by RLAE.

Another requirement is that in the cover letter sent to the editor at the time of submission of the manuscript, a declaration must be included on all previous submissions and reports that may be considered as a redundant publication of the same or of a very similar article. Copies of this material must be included in the documents sent in order to assist the editor in decision-making. This measure aims to control the submission of multipart scientific texts which are not accepted by RLAE.

In times of countless changes in the production and dissemination of scientific knowledge, RLAE's editorial policy will continue to be updated in the face of new demands from Open Science.
